# KIF5B modulates central spindle organization in late-stage cytokinesis in chondrocytes

**DOI:** 10.1186/s13578-019-0344-5

**Published:** 2019-10-16

**Authors:** Huiyan Gan, Wenqian Xue, Ya Gao, Guixia Zhu, Danny Chan, Kathryn S. E. Cheah, Jiandong Huang

**Affiliations:** 1 School of Biomedical Sciences, The University of Hong Kong, Hong Kong, People’s Republic of China; 2 Department of Hematology, Nanfang Hospital, Southern Medical University, Guangzhou, People’s Republic of China; 3 Institute of Synthetic Biology, Shenzhen Institutes of Advanced Technology, Chinese Academy of Sciences, Shenzhen, 518055 People’s Republic of China

**Keywords:** Kinesin-1, Cytokinesis, Chondrocytes, Central spindle

## Abstract

**Background:**

The growth plate is a special region of the cartilage that drives longitudinal growth of long bones. Proliferating chondrocytes in the growth plate, arranged in columns, divide perpendicular to the long axis of the growth plate then intercalate to re-align with parental columns. Which molecular partners maintain growth plate columnar structures and chondrocyte cytokinesis has not been fully revealed. It is reported that kinesin family member 3A (KIF3A), a subunit of kinesin-2, plays an important role in maintaining columnar organization in growth plates via controlling primary cilia formation and cell proliferation.

**Result:**

Here we identify kinesin family member 5B (KIF5B), the heavy chain of kinesin-1, a ubiquitously expressed motor protein for anterograde intracellular transport along the microtubule network, as a key modulator of cytokinesis in chondrocytes via maintenance of central spindle organization. We show that KIF5B is concentrated in the central spindle during cytokinesis in both primary chondrocytes and chondrogenic ATDC5 cells.

**Conclusion:**

The failure of cytokinesis in KIF5B null chondrocytes leads to incomplete cell rotation, disrupting proliferation and differentiation, and results in a disorganized growth plate.

## Introduction

The growth plate is regarded as a functional unit of endochondral ossification. Growth plate dysfunction usually leads to bone abnormality, like bone growth retardation [19, 20, 32]. In the mammalian growth plate, flattened proliferating chondrocytes divide along their cell plane perpendicular to the long axis of the growth plate [8]. After cytokinesis, the two daughter cells rotate and intercalate to re-align in the parental column [8], such that the columnar arrangement is retained as the bone grows longitudinally. The continuous proliferation and differentiation of chondrocytes function as an engine for the longitudinal growth of the long bone. Cell proliferation, cytokinesis and cell intercalation are tightly controlled but the key molecular regulators have not been fully identified.

Kinesin is one of the motor proteins that transport various cargoes along microtubules to specific destinations within the cell. The kinesin superfamily proteins (KIFs) consist of 15 kinesin families and more than 45 members in mammals [16]. Although the motor domain is relatively conserved among KIF families, the tail domain is more diverse for its association with unique cargos in each kinesin. KIF3A, a subunit of the kinesin II motor complex, is required for intraflagellar transport and the formation of primary cilia. It is reported that *Kif3a* mutant chondrocytes show loss of primary cilia, reduced proliferation, defective cell rotation and accelerated differentiation, resulting in disrupted columnar organization in the growth plate and post-natal dwarfism [36]. Another KIF, KIF7, also plays an important role in chondrocyte proliferation and differentiation.

Among all kinesins, kinesin-1 is the first identified one and best characterized. It is composed of two light chains and two heavy chains. The latter, called KIF5, occurs in three forms: KIF5A and KIF5C are neuron specific, whereas KIF5B is ubiquitously expressed [15, 39]. In vivo studies of KIF5B in neurons, pancreatic β-cells and myogenic cells indicate its roles in the transport of organelles, membranous vesicles and other cargoes [5, 15, 37]. Depletion of KIF5B in beta cells in mice results in smaller islet size, increased islet number, high insulin vesicle density in β-cell and subsequently leads to insulin secretion deficiency and glucose intolerance. In skeletal muscle cells, KIF5B plays an important role in transporting α-sarcomeric actin, non-muscle myosin IIB, together with intermediate filament proteins desmin and nestin to the growing tips of the elongating myotubes. Mice with Kif5b conditionally ablated in myogenic cells showed aggregation of actin filaments and intermediate filament proteins in differentiating muscle cells, which subsequently led to defect in myofibril assembly. Although the functions of KIF5B have been intensively studied, the role of KIF5B in chondrocytes remains unknown. To assess the role of KIF5B in growth plate chondrocytes, we established a mutant mouse model with *Kif5b* selectively ablated in chondrocytes (*Col2cre*; *Kif5b*^*fl/*−^ mice). Our results not only demonstrate the role of KIF5B in chondrocyte proliferation and differentiation, but also reveal KIF5B as a significant modulator in chondrocyte cytokinesis.

## Results

### KIF5B depletion in chondrocytes leads to growth plate abnormalities

**Fig. 1:**
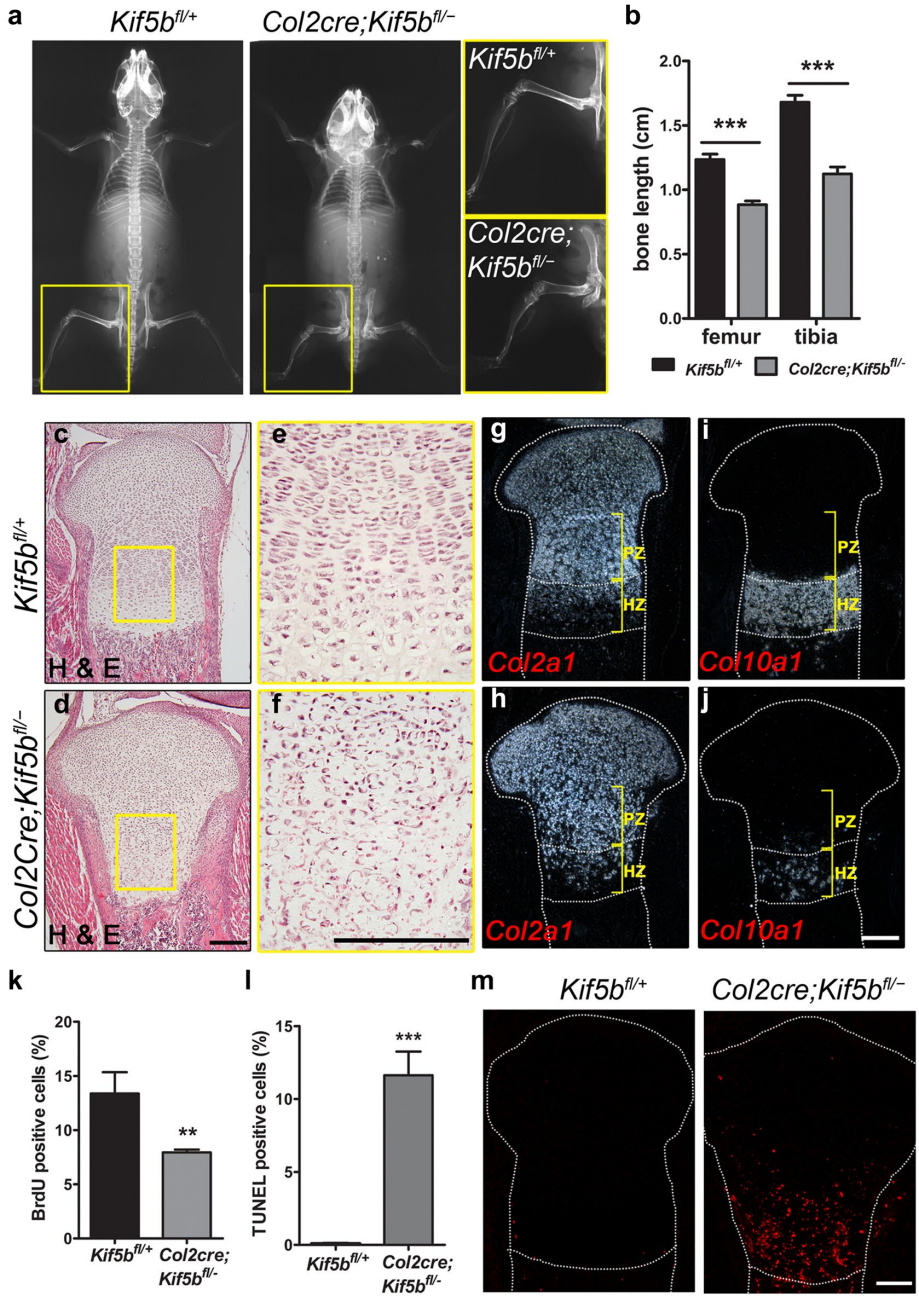
Col2a1-Cre directed depletion of KIF5B leads to growth plate abnormalities. **a** Representative X-ray images of the *Kif5b*^*fl/*+^ and *Col2cre*; *Kif5b*^*fl/*−^ mice at 6 weeks of age. **b** Length of long bones of *Kif5b*^*fl/*+^ and *Col2cre*; *Kif5b*^*fl/*−^ mice at 6 weeks of age (*Kif5b*^*fl/*+^: *n *= 5; *Col2cre*; *Kif5b*^*fl/*−^: *n *= 5). ***P < 0.0001; unpaired two-tailed t-test. Data are shown as mean ± S.D. **c**, **d** H & E staining on sections of the proximal tibial growth plate of P1 newborns. **e**, **f** Enlarged images showing absence of columnar structure the mutant growth plate. **g**–**j** In situ hybridization of *Col2a1* and *Col10a1* probes on adjacent sections for H & E staining. Scale bar (**c**–**j**): 200 μm. **k** Quantification of BrdU-positive chondrocytes in the entire proximal tibial growth plate of P1 newborns (*Kif5b*^*fl/*+^: *n *= 3; *Col2cre*; *Kif5b*^*fl/*−^: *n *= 3). Three sections were counted for each sample. **P = 0.0092; unpaired two-tailed t-test. Data are mean ± S.D. **l** Quantification of TUNEL-positive chondrocytes in the entire proximal tibial growth plate of P1 newborns (*Kif5b*^*fl/*+^: *n *= 3; *Col2cre*; *Kif5b*^*fl/*−^: *n *= 3). Three sections were counted for each sample. ***P = 0.0003; unpaired two-tailed t-test. **m** TUNEL assay on sections of the proximal tibial growth plate of P1 newborns. Scale bar: 150 μm

*Kif5b* mutant mice were viable and had a normal life span, but they were smaller in stature owing to shortened spine vertebrae and long bones (Fig. 1a, b). The whole animal skeletal preparation also confirms that both the forelimbs and the hindlimbs of the mutants are severely shortened at the newborn stage. The skeletal preparation of mice at postnatal day 10 further reveals growth retardation of the long bones (Additional file 1: Figure S1). Safranin O binds to the proteoglycans and glycosaminoglycans in the cartilage, giving the cartilage an orange color. A thick hypertrophic zone could be observed in the control growth plate, whereas only a few hypertrophic chondrocytes could be observed at either side of the cartilage in the mutant plate (Additional file 2: Figure S2). Immunostaining of the long bones revealed that KIF5B protein was depleted in the majority of chondrocytes in the proliferating and hypertrophic zones of the cartilage, although residual KIF5B protein was detectable in the resting chondrocytes (Additional file 3: Figure S3A). *Kif5a* and *Kif5c* expression was not seen in the growth plate in control or mutant mice (Additional file 3: Figure S3B). Therefore there is no compensation from any other KIF5 isoforms in *Kif5b* mutant chondrocytes.

**Fig. 2:**
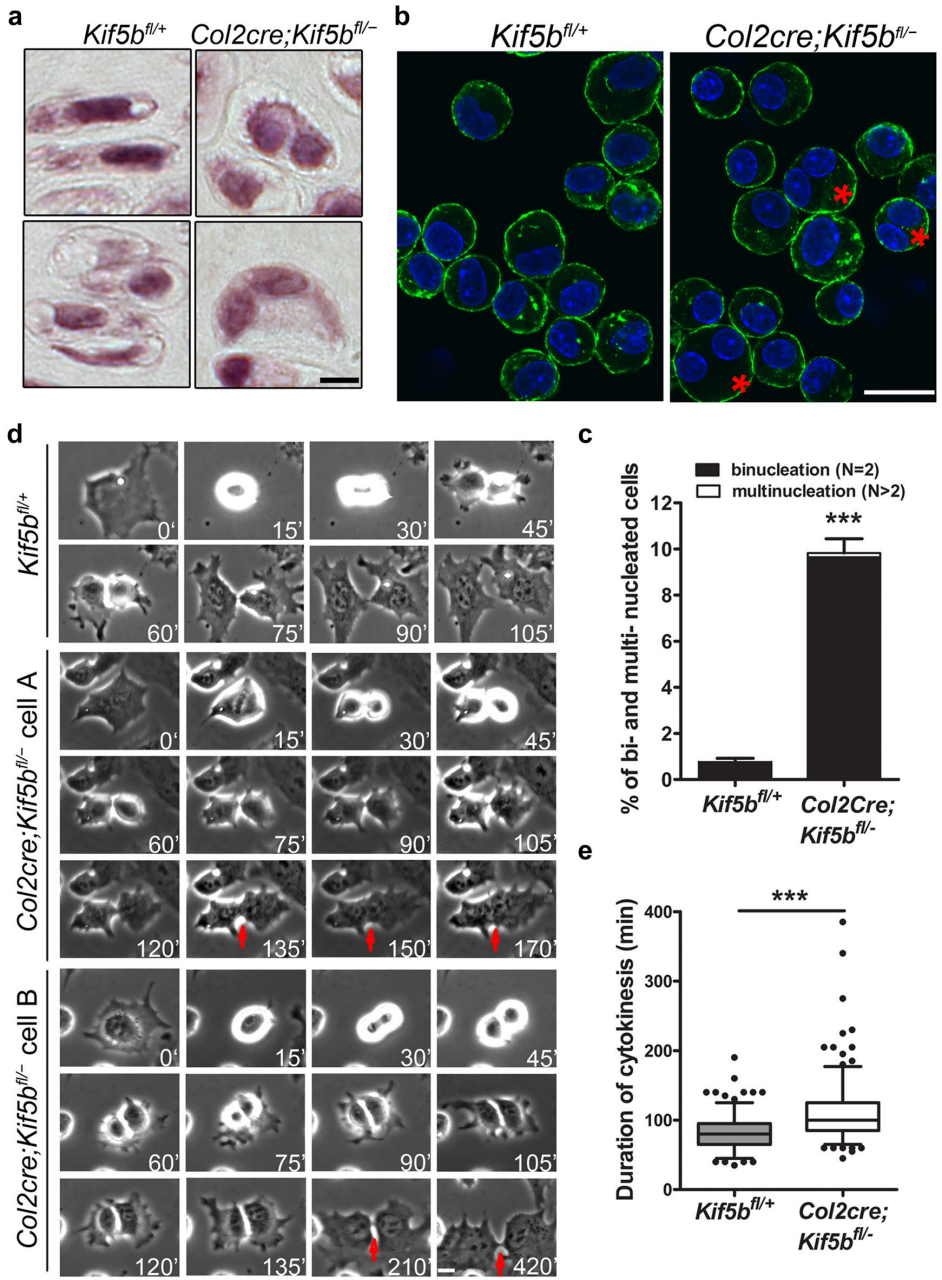
KIF5B deficient chondrocytes display cytokinetic defect. **a** H & E staining on sections of the proximal tibial growth plate of P1 newborns showing binucleated cells in mutant growth plates. Scale bar: 5 μm. **b** Cytospin of isolated chondrocytes from the proliferating zone of tibia and femur of P1 newborns were stained with phalloidin (green) and DAPI (blue). Asterisks denote typical binucleated cells. Scale bar: 20 μm. **c** Quantification of bi- and multi-nucleated rate in cytospin preparation samples from P1 newborns (*Kif5b*^*fl/*+^: *n* = 5; *Col2cre*; *Kif5b*^*fl/*−^: *n* = 5). ***P < 0.0001; unpaired two-tailed t-test. Data are mean ± S.D. **d** Time-lapse images of primary chondrocytes in mitosis. Scale bar: 10 μm. **e** Quantification of cytokinesis duration of primary chondrocytes (*Kif5b*^*fl/*+^ cells: *n* = 232; *Col2cre*; *Kif5b*^*fl/*−^ cells: *n* = 231). ***P < 0.0001; two-tailed Mann–Whitney *U*-test. The whisker plot shows median (lines), interquartile range (boxes) and 5% to 95% percentile (whiskers). Duration of cytokinesis was calculated from the furrow ingression to the final separation of the two daughter cells. Cells fusing back were not included in this analysis

In newborn *Kif5b* mutant mice, the cartilage in the long bones showed gross histological abnormality, characterized mainly by disorganized columnar structure in the growth plates (Fig. 1c–f). In the proliferating zone of controls, chondrocytes adopted a discoid cell shape and were aligned into columns (Fig. 1e). In the mutants, columnar structure was disrupted; chondrocytes were mis-oriented and formed whorl-like cell–cell clusters (Fig. 1f). There were few chondrocytes with the normal hypertrophic chondrocyte morphology (Fig. 1d, f). Whereas a clearly defined hypertrophic zone was observed in control growth plates, in the mutants we detected very few cells expressing the hypertrophic chondrocyte specific marker *Col10a1* in the lower region of mutant growth plates (Fig. 1g–j), suggesting that chondrocyte terminal differentiation is affected in *Kif5b* mutants. Furthermore, we found that chondrocytes in the growth plate of *Kif5b* mutants showed a decreased proliferation accompanied by significantly increased apoptosis (Fig. 1k–m and Additional file 3: Figure S3C). In sections of mutant cartilage, we frequently observed binucleated and multinucleated cells in the growth plates (Fig. 2a): 9.83% of chondrocytes isolated from *Col2cre*; *Kif5b*^*fl/*−^ growth plates compared to 0.76% of those from controls (*P *< 0.0001, *n *= 5/5; Fig. 2b, c), indicating a cytokinetic defect in KIF5B-deficient chondrocytes.

### KIF5B deficiency results in cytokinetic defect in chondrocytes

In *Kif5b* knockout chondrocytes, the cytokinetic defect is one of the most prominent and important phenotypes. We prepared primary cultures of KIF5B-deficient chondrocytes isolated from growth plates and monitored cell division by time-lapse microscopy. The loss of KIF5B in mutant cells was further confirmed by Western Blot analysis and immunofluorescence (Additional file 4: Figure S4A, B). Mutant chondrocytes usually developed normal equatorial furrowing at a similar phase as control chondrocytes (Fig. 2d and Additional file 4: Figure S4, Additional file 5: Movie S1 and Additional file 6: Movie S2). However, they demonstrated a marked delay in abscission (Fig. 2d, e), and 5.2% of dividing cells failed in cytokinesis and fused back instead during the phase of abscission (Fig. 2d) compared to 0.78% of control cells.

**Fig. 3:**
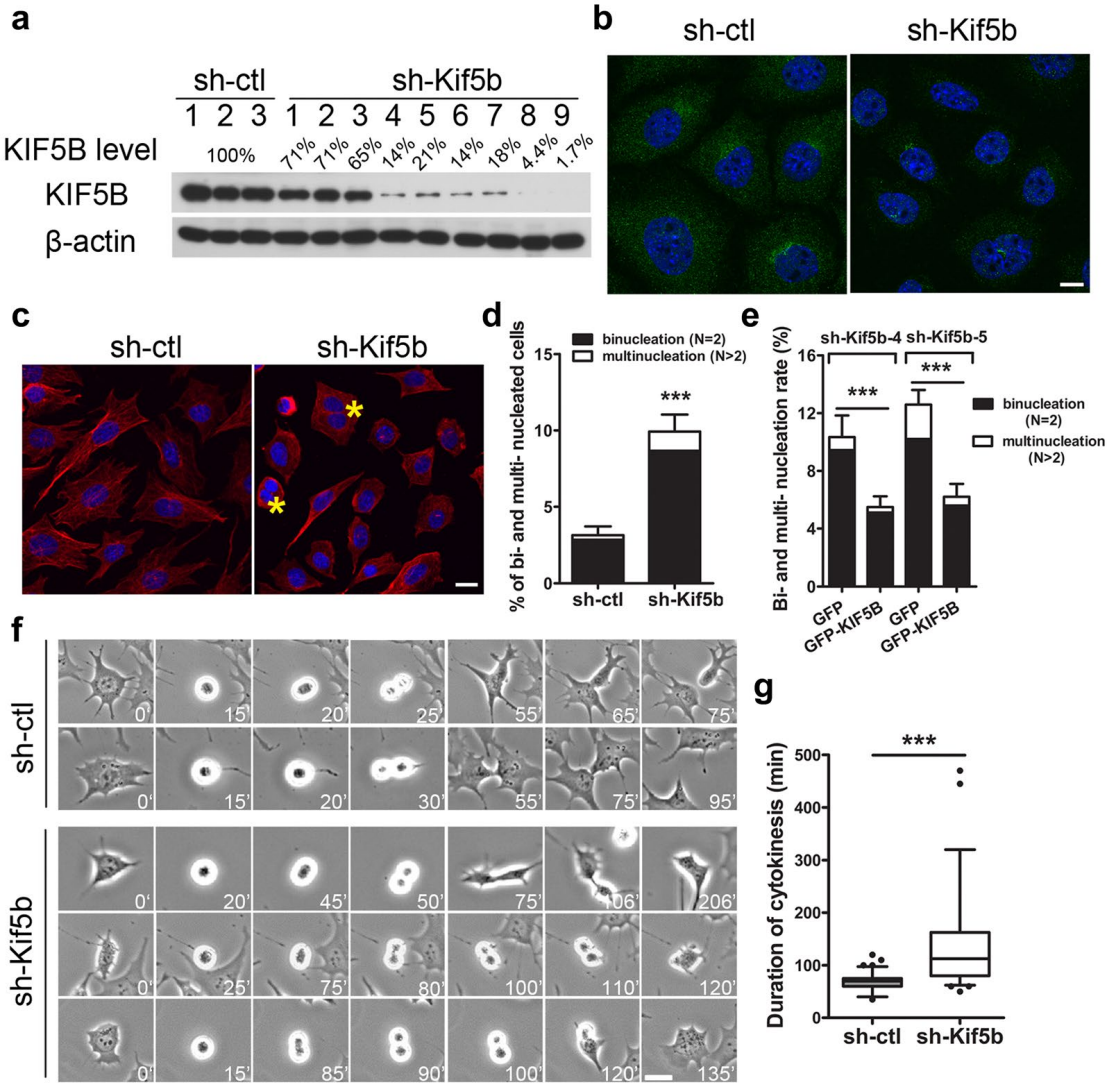
Cytokinetic phenotype in Kif5b knockdown ATDC5 cells. **a** Western blot of protein extracts from single cell clones with stable expression of sh-ctl or sh-kif5b constructs. **b** Immunofluorescence of KIF5B (green) in sh-Kif5b clone #4. Scale bar: 10 μm. **c** sh-ctl and sh-Kif5b cells stained with α-tubulin (red) and DAPI (blue). Yellow asterisks denote typical binucleated cells. Scale bar: 20 μm. **d** Quantification of the bi- and multi-nucleation rate in control cell clones (*n* = 3, sh-ctl clone #1–3) and Kif5b-knockdown cell clones (*n* = 5, sh-Kif5b clone #4–8). ***P < 0.0001; unpaired two-tailed t-test. Data are mean ± S.D. **e** Re-introduced GFP tagged full length KIF5B reduced the bi- and multi-nucleation rate in Kif5b knockdown ATDC5 cells clone #4 (*n* = 10 independent experiments) and #5 (*n* = 5 independent experiments). ***P < 0.0001; unpaired two-tailed t-test. Data are mean ± S.D. **f** Time-lapse images of sh-ctl and sh-Kif5b cells in mitosis. Scale bar: 10 μm. **g** Quantification of duration of cytokinesis in sh-ctl cells (*n* = 84 cells from sh-ctl clone #1–3) and sh-Kif5b cells (*n* = 68 cells from sh-Kif5b clone #4, #5 and #8). ***P < 0.0001; two-tailed Mann–Whitney *U*-test. The whisker plot shows median (lines), interquartile range (boxes) and 5% to 95% percentile (whiskers). Duration of cytokinesis was calculated from the furrow ingression to the final separation of the two daughter cells. Cells fusing back were not included in this analysis

Since prolonged monolayer primary culture can trigger chondrocyte de-differentiation [12], we performed further in vitro study in a widely used chondrogenic cell line, ATDC5 [34]. *Kif5b* was knocked down in ATDC5 cells using shRNA and stably knocked down cell lines were established from single cell clones. The frequency of bi- or multi-nucleation was significantly increased in the clones with KIF5B protein reduced to 4% to 20% of normal level (clone #4–#8) (Fig. 3a–d). Re-introduction of GFP-tagged full-length *Kif5b* cDNA into the knockdown cells reduced the percentage of binucleation (Fig. 3e). The time-lapse videos for sh-Kif5b clones #4, #5 and #8 showed that overall 7.5% of *Kif5b*-knockdown dividing cells failed in cytokinesis and fused back to become binucleated during abscission (Fig. 3f). The *Kif5b* knockdown cells also required a significantly longer time for abscission (Fig. 3g).

**Fig. 4:**
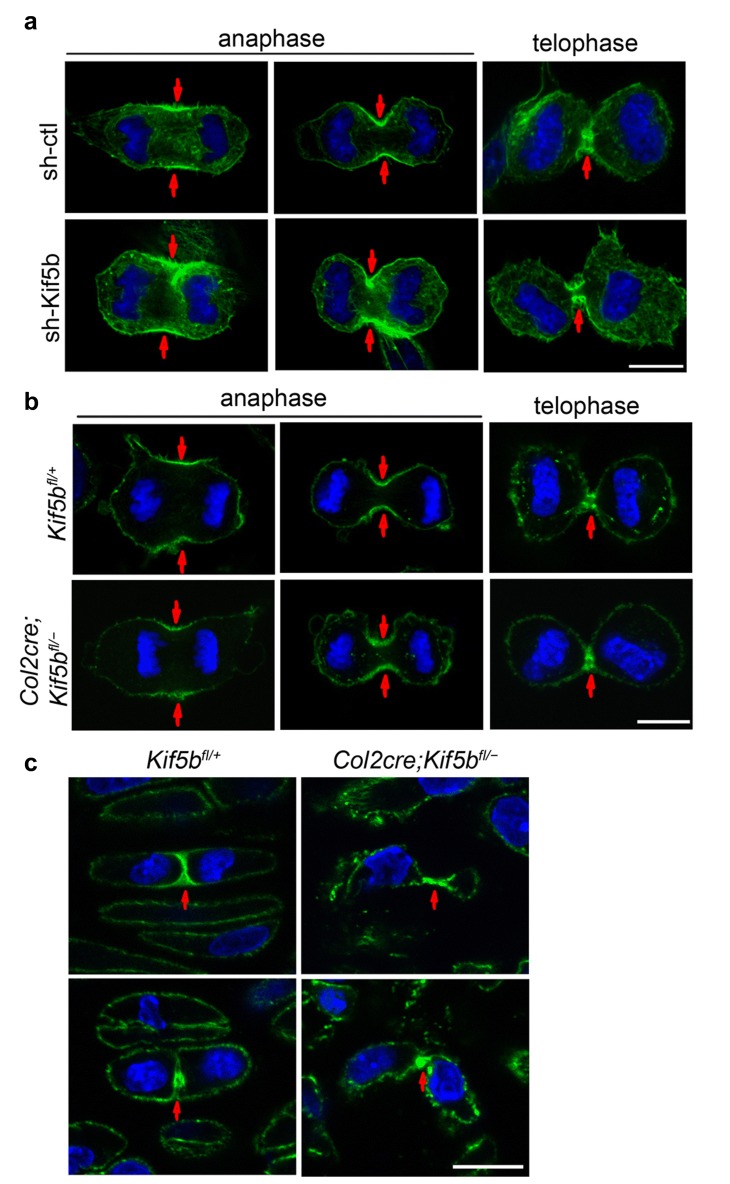
Formation of contractile ring is not affected in KIF5B deficient chondrocytes. **a** sh-ctl and sh-Kif5b ATDC5 cells stained with phalloidin (green) and DAPI (blue). Scale bar: 10 μm. **b** Primary chondrocytes stained with phalloidin (green) and DAPI (blue). Scale bar: 10 μm. **c** Frozen sections of proximal tibial growth plate of P1 newborns stained with phalloidin (green) and DAPI (blue). Scale bar: 10 μm. Red arrows denote the cleavage furrow in the dividing cells

As a marker of cytokinesis onset, cleavage furrow formation is triggered by the contraction of actin filaments on the equatorial cortex [6]. Time-lapse videos (Figs. 2d and 3f) showed that in *Kif5b*-deficient cells, the cleavage furrow formed properly during early cytokinesis. Phalloidin staining showed that F-actin was enriched at the equatorial cortex during early cytokinesis in both the control and *Kif5b* knockdown cells (Fig. 4a). Similarly, the highest concentration of F-actin was at the cleavage furrow in control and *Kif5b* mutant chondrocytes in vivo and in vitro (Fig. 4b, c). Hence, the formation of the cleavage furrow and the contractile ring in early cytokinesis are not affected by the depletion of KIF5B, however, cytokinesis fails to continue at later stages after the narrow intercellular bridge had been formed. In normal proliferative chondrocytes, Golgi apparatus (marked by GM130 with red luorescence) localizes to one or two sides of the nucleus (marked with DAPI). The cell plane is perpendicular to the longitudinal axis of the growth plate (Additional file 7: Figure S5A upper). But in KIF5B depleted chondrocytes, the cells are abnormal shaped. Golgi complex scatters around the cytoplasm, with the cell planes abnormally aligned, compared to the longitudinal axis of the growth plate (Additional file 7: Figure S5A lower). It is shown that most normal cells display cilia when stained with the antibody for acetylated-α-tubulin (Additional file 7: Figure S5B upper). As well, cilia are preferentially located on the inferior/superior surfaces of the lattened chondrocytes. However, although the epiphyseal chondrocytes in the mutant growth plate are less afected, the proliferative chondrocytes are nearly devoid of cilia, with the acetylated tubulin scattered in the whole cell (Additional file 7: Figure S5B lower).

### Central spindle is defective in KIF5B deficient chondrocytes in late cytokinesis

**Fig. 5:**
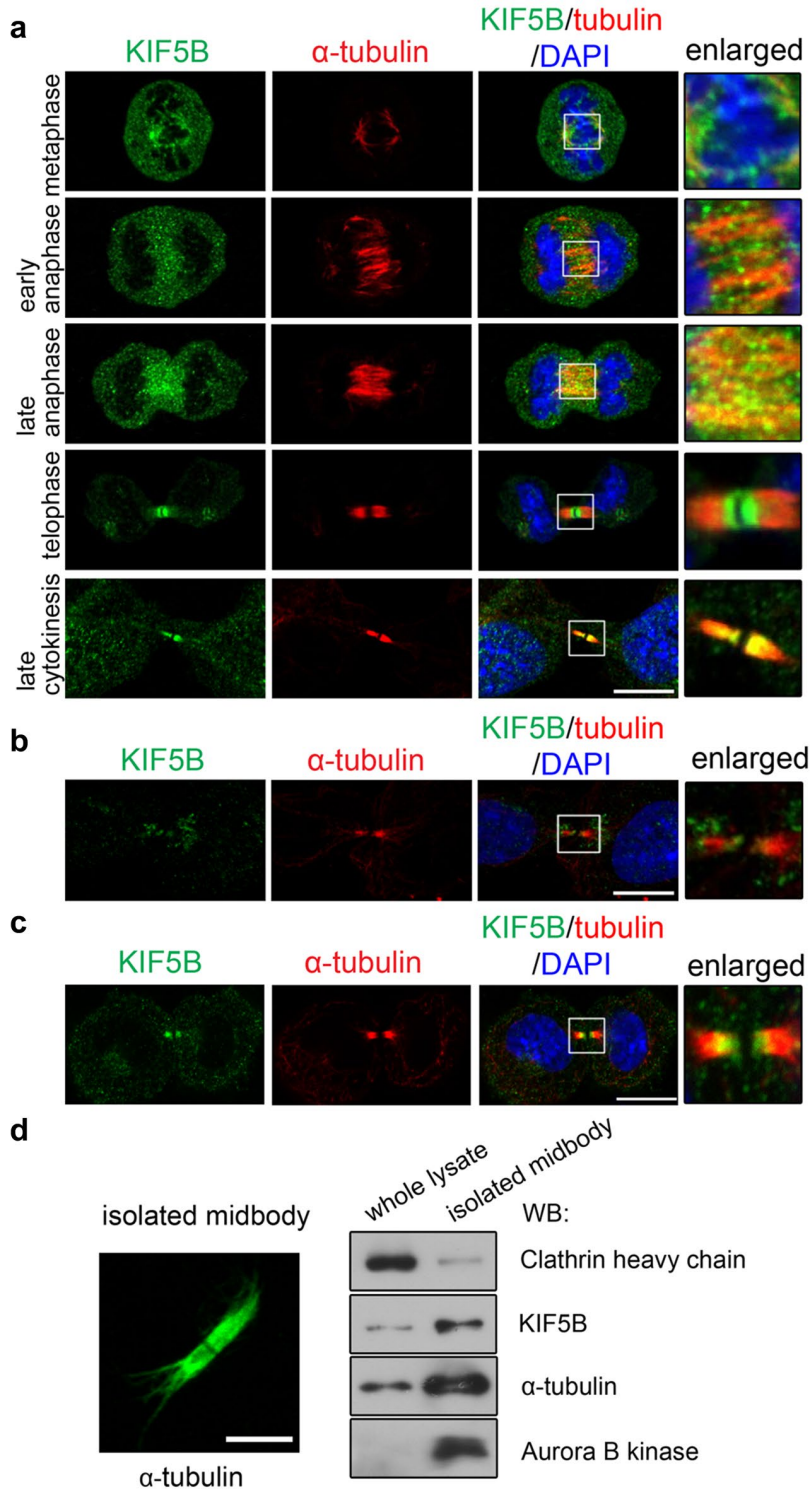
KIF5B localizes to central spindle during cytokinesis in chondrocytes. **a** Immunofluorescence of KIF5B (green) and α-tubulin (red) in ATDC5 cells in different phases of mitosis. Scale bar: 10 μm. **b** Immunofluorescence of KIF5B (green) and α-tubulin (red) in sh-Kif5b knockdown ATDC cells (clone #4) in cytokinesis. Scale bar: 10 μm. **c** Immunofluorescence of KIF5B (green) and α-tubulin (red) in primary chondrocytes in late cytokinesis. Scale bar: 10 μm. **d** Midbody isolation using ATDC5 cell lines. Immunofluorescence of α-tubulin showing the isolated midbody (left panel). Scale bar: 5 μm. Western Blot showing that KIF5B, α-tubulin and Aurora B kinase were present in midbody fraction (right panel)

The central spindle is assembled in anaphase and maintained until the late stage of cytokinesis. Prolonged and failed cytokinesis is frequently associated with abnormal central spindle structures. Immunostaining showed that in metaphase KIF5B partially localized to the mitotic spindle (Fig. 5a, metaphase); while in anaphase KIF5B was gradually concentrated in the mid-zone (Fig. 5a, anaphase). With further ingression of the cleavage furrow, KIF5B was enriched in the central spindle, which is usually referred to as the midbody in late cytokinesis (Fig. 5a, telophase and late cytokinesis). The localizations of KIF5B observed here were specific, since the signals of KIF5B in the sh-Kif5b cells were largely reduced in both cytoplasm and the midbody region (Fig. 5b). The same pattern of KIF5B localization at midbody was observed in primary chondrocytes (Fig. 5c) and the isolated midbody fraction in ATDC5 cells (Fig. 5d).

**Fig. 6:**
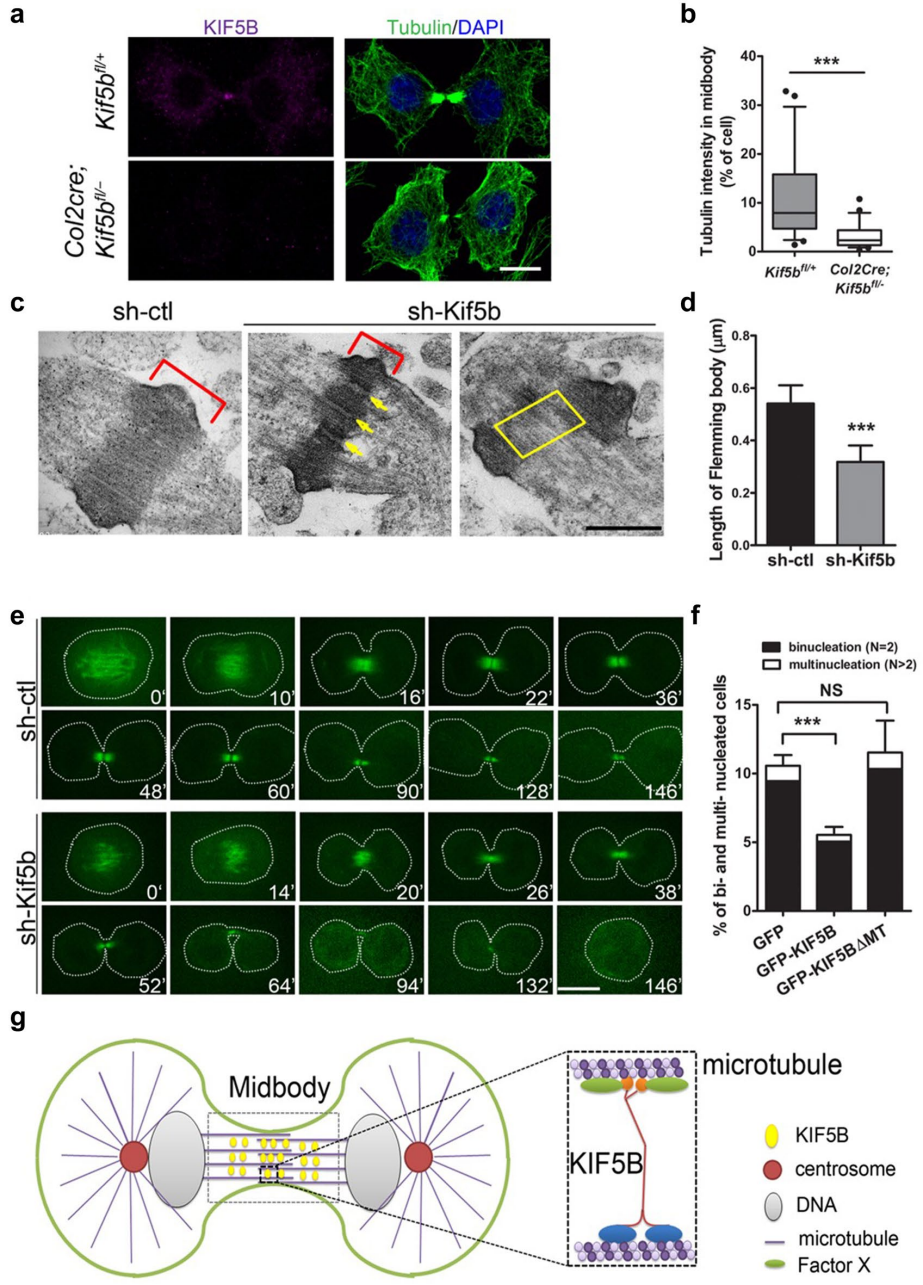
Midbody microtubule organization is impaired in Kif5b deficient cells. **a** Immunofluorescence of α-tubulin in primary chondrocytes in late cytokinesis. Scale bar: 10 μm. **b** Quantification of tubulin intensity in midbody in primary chondrocytes (*Kif5b*^*fl/*+^ cells: *n* = 24; *Col2cre*; *Kif5b*^*fl/*−^ cells: *n* = 27). ***P < 0.0001; two-tailed Mann–Whitney *U*-test. The whisker plot shows median (lines), interquartile range (boxes) and 10% to 90% percentile (whiskers). **c** Electron micrographs of midbody regions from sh-ctl and sh-Kif5b ATDC5 cells. Red lines denote the region of Flemming body. Yellow arrows and boxes denote the broken regions in Flemming body. Scale bar: 0.5 μm. **d** Quantification of the length of Flemming body in sh-ctl and sh-Kif5b cells (sh-ctl cells: *n* = 12; sh-Kif5b cells: *n* = 9). ***P < 0.0001; unpaired two-tailed t-test. Data are mean ± S.D. **e** Live imaging of sh-ctl and sh-Kif5b cells expressing GFP-tubulin in cytokinesis. Scale bar: 10 μm. **f** Quantification of the bi- and multi-nucleation rate in sh-Kif5b cells transiently expressing GFP, GFP-Kif5b and GFP-Kif5bΔMT (*n* = 5 independent experiments). ***P < 0.0001; NS, *P *= 0.4039; unpaired two-tailed t-test. Data are mean ± S.D. **g** A model showing the function of KIF5B in cytokinesis. In late cytokinesis, KIF5B alone or together with other unknown molecules cross-links microtubules in the midbody, and therefore the structure of midbody can be stably maintained

The localization of KIF5B to the midbody in late cytokinesis is similar to other cytokinesis-related motors such as KIF4 and MKLP1 [21, 44], suggesting that KIF5B may have central spindle-related functions. Further analysis of midbody markers and structure showed that tubulin concentration in the midbody was reduced in *Kif5b*-deficient primary chondrocytes and in *Kif5b* knockdown ATDC5 cells (Fig. 6a, b and Additional file 8: Figure S6A, B). However, the localization of mitotic kinesin-like protein (MKLP1) and citron kinase (CIT-K), two key components of the midbody, was not affected (Additional file 9: Figure S7). Observation of the midbody ultrastructure by electron microscopy showed that the midbody matrix (or Flemming body) [9], a stable dense structure for gluing local microtubules in that region [27], was thinner or broken in *Kif5b* knockdown cells (Fig. 6c, d and Additional file 10: Figure S8). Disorganization or absence of this structure is related to cytokinetic failure [2, 23, 42]. In the *Kif5b* knockdown cells, the microtubule bundles within the midbody region appeared sparser and less compact than that in control cells (Additional file 10: Figure S8). Live imaging of GFP-tubulin labeled *Kif5b* knockdown cells showed that the central spindle was defective and not well maintained during late cytokinesis, although it was normally assembled in early cytokinesis (Fig. 6e lower panel and Additional file 11: Movie S3, Additional file 12: Movie S4).

**Fig. 7:**
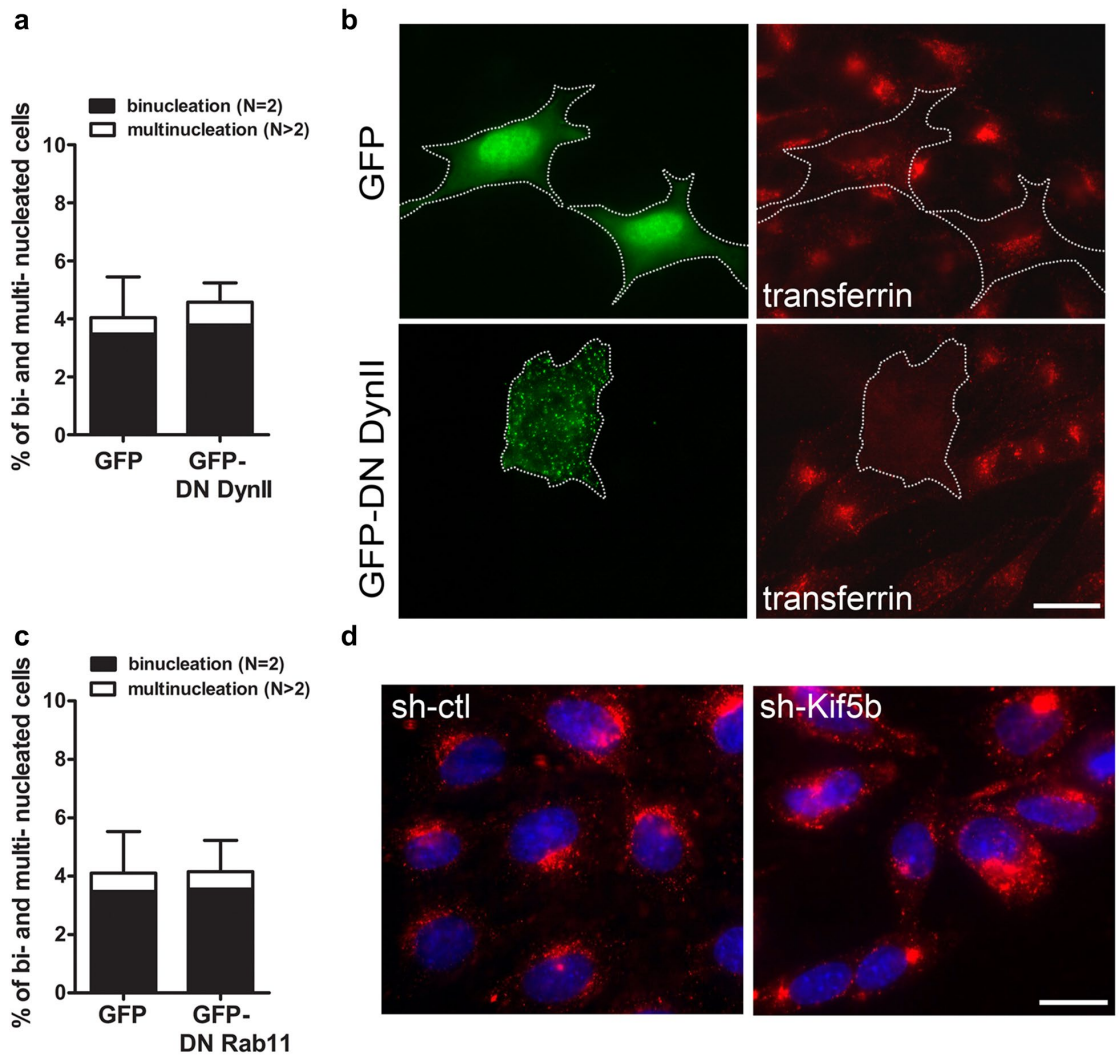
Inhibition of endocytosis and endosomal trafficking does not have a significant effect in cytokinesis in ATDC5 cells. **a** Quantification of bi- and multi-nucleation rate in GFP and GFP-dynamin 2 K44A positive cells (*n* = 5 independent experiments). *P *= 0.4373; unpaired two-tailed t-test. Data are mean ± S.D. **b** Absence of transferrin (red) uptake in GFP-dynamin 2 K44A positive cells (green) showing inhibition of clathrin-dependent endocytosis. Scale bar: 20 μm. **c** Quantification of bi- and multi-nucleation in GFP and GFP-Rab11 S25N positive cells (*n* = 5 independent experiments). *P *= 0.8207; unpaired two-tailed t-test. Data are mean ± S.D. **d** Transferrin (red) uptake in sh-ctl and sh-Kif5b ATDC5 cells. Scale bar: 20 μm

The completion of cytokinesis, marked by the cleavage of the intercellular cytoplasmic bridge, requires delivery of membranous vesicles to the midzone. Inhibition of clathrin-dependent endocytic and recycling endocytic pathways leads to a high binucleation rate in Hela cells [26, 33, 38, 41]. One study showed that KIF5B participates in transporting the recycling endosomal vesicles to the midzone in Hela cells [26]. Dynamin 2 contributes to the formation of clathrin-coated vesicles by constricting and cutting the coated vesicles off the plasma membrane [7], and overexpression of mutant dynamin 2 specifically inhibits clathrin-mediated endocytosis [22, 30]. However, we found that over-expression of dominant negative dynamin 2 did not significantly induce binucleation in ATDC5 cells even though it inhibited clathrin-dependent endocytosis (Fig. 7a, b). Blocking the recycling endosomal pathway by over-expressing a dominant mutant of Rab11 (Rab11S25N) also had no effect on cytokinesis in ATDC5 cells (Fig. 7c). In addition, we did not observe any significant inhibition of clathrin-dependent endocytosis in the *Kif5b*-knockdown ATDC5 cells (Fig. 7d). Therefore, unlike in Hela cells, neither clathrin-mediated endocytosis nor recycling endosomal trafficking seems to be significantly involved in the cytokinesis of chondrogenic ATDC5 cells, thus KIF5B must play a role in cytokinesis through mechanisms other than endocytic membrane vesicle trafficking.

### KIF5B stabilizes central spindle in late cytokinesis

Next, we investigated which domain of KIF5B protein is important in cytokinesis. Residues 904–915aa are known to be a conserved microtubule binding region of the KIF5B tail [13], which is proposed to be involved in microtubule sliding in a variety of cells in interphase [18]. Over-expression of full length KIF5B but not Kif5b-ΔMT (without residues 904–915aa) reduced the amount of bi- and multi-nucleation in Kif5b knockdown ATDC5 cells (Fig. 6f). Therefore, we propose that KIF5B may function in maintaining the central spindle through microtubule cross-linking in late cytokinesis, possibly functioning via its microtubule-binding site (residues 904–915aa) (Fig. 6g). However, we cannot rule out that some other molecules may act in concert with KIF5B in this process. A recent report showed that purified kinesin-1 from porcine brain failed to bundle taxol-stabilized microtubules, but the in vitro bundling activity was evident in the presence of NudC protein [40]. Therefore, it is possible that the tail region in KIF5B is involved in recruiting other protein partners to stabilize the central spindle in late cytokinesis (Fig. 6g).

## Discussion

Kinesin members are known to have multiple functions in cytokinesis. Some play a role in transporting membrane vesicles to cleavage furrow while some are responsible for transporting important components to midbody. A report shows that KIF5B, together with KLC and JIP4, transports recycling vesicles to the cleavage furrow during late cytokinesis in Hela cells [26]. However, loss of KIF5B only prolongs abscission time, but does not cause cytokinetic failure [26]. Until now, there are no other reports on KIF5B’s functions in relation to cytokinesis in any other cell types. Another kinesin, KIF3B, was found participating in cytokinesis by delivering syne-1-associated vesicles to the cleavage furrow [10]. KIF13A serves to translocate a PtdIns(3)P-binding protein, FYVE-CENT to midbody [31]. Besides, KIF4, a chromokinesin, travels to midzone during early anaphase with nonmotor microtubule associated protein PRC1, and promotes the central spindle assembly [21]. Kinesin-6 family proteins MKLP1 and MKLP2 also translocate important central spindle components to the midzone, such as CYK4, Plk1, and Aurora B kinase [25, 28].

Other than the functions of trafficking, several kinesin proteins are also able to bundle microtubules in midzone. In vitro microtubule binding and bundling experiments indicate that MKLP2, MMP1, MKLP1, and CHO1 (an isoform of MKLP1) have strong capability of bundling microtubules [23, 28, 29]. Abrogation of these protein functions in vivo usually leads to malformed central spindle and failure in cytokinesis. However, how these molecules cross-link microtubules remains unknown. One possible explanation is that more than one microtubule-binding site exist in these motors. For example, MKLP2 has two independent two microtubule-binding sites: one in the N-terminal kinesin motor domain and another at the C-terminus [28]. Similarly, CHO1 appears to have two microtubule-binding sites, both located at the kinesin motor domain [23]. KIF5B has two microtubule-binding sites: one in the N-terminal motor domain and the other in the tail domain [13]. Intriguingly, KIF5B is also able to cross-link microtubules and perform microtubule sliding in the interphase cells [18]. Given the cross-linking capability and our current results, KIF5B could participate in central spindle organization with its motor domain and tail microtubule binding domain during cytokinesis.

Most studies on cytokinesis are based on Hela cells and studies in chondrocytes are rare. To date, two studies on cytokinesis in chondrocytes were reported, one on profilin 1 and the other on β1-integrin. In the study of profilin 1, a mouse model was generated with profilin 1 conditionally knock out in chondrocytes [3]. The mutant mice developed progressive dwarfism at the postnatal stage due to the decreased size of the proliferating zone and the reduced growth rate of the proliferating chondrocytes [3]. The significantly higher binucleation rate in the mutant growth plate was only observed in mutant mice at 4-weeks [3]. Further time-lapse experiment and immunostaining showed that these mutant chondrocytes could form cleavage furrow and assemble the actomyosin contractile ring properly [3]. However, they failed in abscission due to the lack of traction forces for fission [3]. As profilin is a key regulator of actin filament rearrangement, the absence of profilin results in abnormal F-actin distribution and loss of stress fibers in the mutant cells. Besides, focal adhesion is also impaired in mutant chondrocytes, which also contributes to the failure in generating traction forces [3]. The other study showed that β1-integrin deficient chondrocytes have a higher binucleation rate than the controls [1]. The primary mutant chondrocytes appear to have reduced adhesion on different kinds of extracellular matrix [1]. Similar to profilin 1 depleted chondrocytes, they have thinner and fewer actin stress fibers and impaired focal adhesions [1]. Although the mechanisms of the cytokinetic defect have not been further revealed in the study, it is likely that the defect of cytokinesis in the integrin mutant chondrocytes is partially caused by the insufficient traction forces. Altogether, these two studies emphasize the importance of traction forces and cell–matrix interaction during abscission in chondrocytes. However, other aspects of cytokinesis in chondrocytes, such as central spindle formation and maintenance, remain ambiguous. Therefore, our study is the first report that highlights central spindle organization in chondrocyte cytokinesis, and it is also the first evidence that identifies KIF5B as one of the important modulators in central spindle maintenance.

In the growth plate of *Kif5b* mutant mice, the daughter cells may start the cell rotation under the traction of extracellular matrix. However, without complete cell abscission, they may not finish cell rotation and intercalation process, thereby disrupting the columnar structure in the growth plate. We have thus shown that cytokinesis is essential for the maintenance of growth plate columnar structure, and that KIF5B is an important modulator of central spindle organization in late-stage cytokinesis in chondrocytes. Besides, cytokinetic failure may also relate to the reduction of proliferation in chondrocytes as the failure of cell abscission may inhibit the next round of proliferation of the daughter cells. Unlike the reported mitotic motor kinesins such as KIF4 and MKLP1 that have important roles in midbody assembly [21, 43], KIF5B functions in midbody organization and maintenance specifically in late-stage cytokinesis. This study provides new insight into the mechanisms of central spindle maintenance in late-stage cytokinesis. Previously, another two kinesin members, KIF3A and KIF7, were studied in the growth plate chondrocytes. *Kif7 null* chondrocytes show reduced proliferation without any cytokinetic defect [17] whilst KIF3A functions in primary cilium formation and coordinates Wnt and hedgehog signaling pathways, but not in cytokinesis in growth plate chondrocytes [20, 36]. Thus our data reveal the first kinesin that is essential for cytokinesis in chondrocytes. KIF5B deficiency does not result in cytokinetic defect in neuroblastoma cells, myogenic cells or pancreatic cells [5, 37], implying that the molecular motors controlling central spindle organization vary among different cell types. The comparison of kinesin motors in cytokinesis among different cell types will be subjected to future investigation.

KIF5B may have some cytokinesis independent roles in the chondrocytes. We have reported that the secretion of extracellular matrix proteins was affected in the mutant [14]. We also observed that the disruption of terminal differentiation of the chondrocytes and cell death in the mutant growth plate, which may not be directly related to the cytokinetic defect. We are analyzing the details of the phenotypes and may report in a separate article in the future.

Whole-exome sequencing associates missense mutations of a kinesin family member, KIF22, with an autosomal-dominant skeletal dysplasia called Spondyloepimetaphyseal dysplasia with joint laxity [4, 24]. Loss of KIF5B in the cartilage leads to growth plate abnormalities, bone growth retardation and dwarfism, reminiscent of human skeletal dysplasia in mice, which suggests a potential connection between kinesin-1 and human skeletal diseases. Our work warrant further study for clarification of the association in human.

## Conclusion

In summary, we have shown that ablation of Kif5b in chondrocytes results in delayed and defective cytokinesis. In KIF5B-deficient chondrocytes, cleavage furrow specification and ingression proceed normally, but the central spindle structure is not well maintained in late cytokinesis. Furthermore, cell abscission may delay or fail.

## Materials and methods

### Animals

All the breeding and experimental protocols were approved by the Committee on the Use of Live Animals in Teaching and Research in The University of Hong Kong (CULATR #1022-04 and #2046-09). All of the transgenic mice and knockout mice were maintained on a common C57BL/6N genetic background. The *Col2a1*-*cre* mouse line was kindly provided by Dr. Richard R. Behringer (University of Texas, USA). Firstly *Col2a1*-*cre* mice were crossed with heterozygous *Kif5b* null mice to generate *Col2a1*-*cre*; *Kif5b*^+*/*−^ mice. Afterwards these mice were crossed with *Kif5b*^*fl/fl*^ mice, and the *Col2a1*-*cre*; *Kif5b*^*fl/*−^ (*Col2cre*; *Kif5b*^*fl/*−^) mutant mice were generated in 25% probability. Genotyping method is the same as described before [5, 37].

### Antibodies and dyes

KIF5B antibody is homemade and has been described elsewhere (WB: 1:2000; immunofluorescences: 1:250) [5]. Other antibodies were as follow: anti-actin (Sigma, WB 1:2000), anti-α-tubulin (Sigma, WB: 1:10,000; immunofluorescences: 1:2000), anti-Aurora B (BD transduction, WB: 1:1000; immunofluorescence: 1:100), Anti-clathrin heavy chain (BD transduction, WB: 1:1000; immunofluorescence: 1:100), Anti-GM130 (BD transduction, WB: 1:1000; immunofluorescence: 1:100), Anti-PRC1 (Abcam, WB: 1:1000; Santa cruz, immunofluorescence: 1:100), phalloidin conjugated with Alexa Fluor 488 (Molecular probes) for F-actin labeling (1:300), CellLight™ Tubulin-GFP BacMam 2.0 (Molecular probes) for labeling tubulin in live cells, transferrin from human serum conjugated with rhodamine (Molecular probes). For Western Blot secondary antibodies were from Zymed^®^ Laboratories. For immunofluorescence, the secondary antibodies conjugated to Alexa 488, 555, 649 were from Jackson ImmunoResearch Laboratories or Molecular probes (Additional file 8: Movie S1).

### Plasmids and constructs

GFP-dynamin 2 K44A plasmid and GFP-rab11 DN plasmid were purchased from Addgene (#22301 and #12678). They were from Dr. Pietro De Camilli (Yale University, USA) and Prof. Richard Pagano (Minnesota, USA), respectively. The pSilencer TM 3.1-H1 hygro siRNA expression vector containing typical siRNA template insert was a component of commercial kit of Roche, in which, this vector was used as sh-ctl construct. To generate sh-Kif5b constructs, complementary annealed shRNA oligos targeting mouse Kif5b GCCTTATGCCTTTGATCGT were inserted into the pSilencer™ 3.1-H1 hygro siRNA plasmids digested with *Bam*H1 and *Hin*dIII. The GFP tagged Kif5b rescue constructs containing the sequence coding for Kif5b residues GCCTTATGCCTTTGATCGT was rendered knockdown-proof using silent mutations (to give GCCTTATGCgTTcGAcCGT). The Kif5b full length or truncated fragments were ligated into pEGFP-C1 vector between *Kpn*I and *Bam*H1 sites (Additional file 9: Movie S3).

### Primary culture of chondrocytes

For primary chondrocyte culture, the growth plates of femur and tibia of newborn mice were isolated with blade and fine forceps. Perichondrium was removed from the cartilages and growth plates mainly with the proliferating zones were dissected out and digested in DMEM with 2% FBS, 2 mg/mL collagenase 2 and 100 U/mL penicillin and streptomycin in the cell culture incubator for 3 h at 37 °C incubator under 5% CO_2_ in Petri dish. Then 40 μm cell strainers were used to remove remnant cartilages. Chondrocytes were collected by centrifugation. The cells were then washed with PBS twice and resuspended in DMEM growth medium, and then culture on fibronectin-coated surface at 37 °C incubator under 5% CO_2_. For cytospin preparation, freshly isolated chondrocytes were resuspended in 80 μL cytospin buffer (145 mM NaCl, 2.7 mM EDTA, 5% BSA, pH7.3) and applied to the vials for centrifugation (Shandon cytocentrifuge) at 500*g* for 5 min. The slides were air dried before staining (Additional file 10: Figure S6; Additional file 11: Figure S7; Additional file 12: Figure S8).

### Transfection into ATDC5 cells

The ATCD5 cell line was kindly provided by Dr. Chisa Shukunami (Kyoto University). ATDC5 cells were maintained in DMEM/F12 medium containing 5% FBS supplemented with 3 × 10^−8^ M sodium selenite, 10 μg/mL transferrin. Plasmids DNA were transfected into ATDC5 cells using FuGene 6 or FuGene HD transfection reagent (Roche) according to the manufacturer. For siRNA stable knocked down clones, the pSilencer TM 3.1-H1 hygro siRNA expression vectors containing siRNA targeting mouse Kif5b or control were first transfected into ATDC5 cells and then were cultured in growth medium containing 300 μg/mL hygromycine for 2 week. Single cell clones were collected and sub-cultured for further study.

### Midbody isolation

When ATDC5 cells reached 60–70% confluence they were cultured with growth medium with 50–100 ng/mL nocodazole for 14 h. After that the medium was removed and the cells were washed with warm PBS for twice. The cells in metaphase were shaken off from the dishes and collected by centrifugation at 500*g* for 5 min. The cell pellets were resuspended in 10 mL pre-warmed growth medium and cultured at 37 °C for 1.5 h to enter cytokinesis. The cells were collected and then midbodies were isolated in a taxol and phalloidin-containing medium to stabilize the midbody structure as described [35]. Following lysis in a hypotonic buffer that included Triton X-100, insoluble midbodies were pelleted at 2000×*g* in 40% glycerol.

### Transmission electron for midbody examination

Ten 10 cm culture dishes of ATDC5 cells were used for one experiment. The cells were synchronized by nocodazole treatment, and the cells in metaphase were shaken off and collected by centrifugation. They recovered in fresh growth medium and entered cytokinesis. When the cells in cytokinesis phase were enriched, they were collected and fixed in 1 mL 2.5% glutaraldehyde in cacodylate buffer (0.1 M sodium cacodylate-HCl buffer pH 7.4) for 1 h at 4 °C. The cells were centrifuged and resuspended in 1 mL cacodylate buffer with 0.1 M sucrose and kept at 4 °C overnight. On the next day, the cells were washed with cacodylate buffer for twice and then fixed again with OsO_4_ for 30 min at RT. The cells were washed with cacodylate buffer and then embedded into 2% agar gel blocks. The gel blocks were cut into several 1 mm cubes. They were dehydrated with a series of ethanol solution and then incubated with propylene oxide and later infiltrated with epoxy resin/propylene oxide 1:1 mixture for overnight at 37 °C. After that the cell cubes were infiltrated with epoxy resin for 1 h and 30 min at 37 °C with vacuum and embedded with epoxy resin in the molds. The EM sectioning was carried out in the Electron Microscopy Unit in Queen Mary Hospital. The ultrathin sections were examined under a Philips EM208s transmission electron microscope in the EM unit.

### Histological methods

Paraffin sections were prepared as standard method and were processed to H & E staining. In brief, the samples were fixed in 4% paraformaldehyde overnight, decalcified in 0.5 M EDTA for another overnight and dehydrated with a series of ethanol solutions and finally embedded in paraffin and sectioned for histological evaluation. Hematoxylin and eosin staining was performed using standard techniques. For preparing frozen sections, hindlimb samples were fixed in 4% PFA for 4 h and infiltrated with 30% sucrose at 4 °C overnight, and embedded in OCT medium. Cryosectioning was performed in the cryostat (CM 1900UV, Leica), and the sections were cut at 7–15 μm thick and processed for immunofluorescence.

### In situ hybridization on paraffin sections

In situ hybridization on dewaxed sections was performed as previously described [11], using digoxigenin-labelled ribopobes for Col10a1 and Col2a1 [11].

### Immunohistochemistry and immunofluorescence

Immunohistochemistry were performed as DAKO manufacture instructed. TUNEL assay was performed according to the instruction manual of In Situ Cell Death Detection Kit (Roche). BrdU staining was performed according to the BrdU staining Kit (Invitrogen). For cell immunofluorescence, cells were grown on round glass coverslips (13 mm) coated with 10 μg/mL fibronectin. The cells cultured on coverslips or cytospinned onto slides were fixed in 4% PFA for 15 min at RT or cold methanol for 5 min for immunofluorescence. When fixed with PFA, cells were then permeabilized with 0.25% Triton X-100. After blocking, cells were incubated with primary antibodies at 4 °C overnight and then with secondary antibody for 30 min at room temperature. At last cells were mounted in Slowfade^®^ Gold antifade reagent with DAPI (Molecular Probes).

### Microscopy

Images were captured using an Olympus BX51 microscope or Carl Zeiss LSM700 (Germany) laser scanning confocal microscope with a Plan Apochromat 20× or 63× 1.4NA oil immersion objective. Wide-field images were taken by Spot RT3 Digital Camera and Spot advanced software. Confocal images were collected with ZEN software (Carl Zeiss, Germany) and analyzed with Metamorph (Version 7.7.11, Molecular Device, US) and Image J software. For bright field live cell imaging, we used a ZEISS Axio Observer microscope (Carl Zeiss, Germany) equipped with an environmental control chamber. Confocal live imaging was performed using a Perkin-Elmer UltraView ERS spinning disk system (PerkinElmer Inc.) attached to a ZEISS Axio Observer microscope with an EM CCD camera (Evolve512, Photometrics, US). Images were processed with MetaMorph software (Version 7.7.11, Molecular Device, US).

### Quantitative and statistical analysis

All statistical analysis was performed using the GraphPad Prism software (version 5.00; GraphPad Software). Student’s t test or Mann–Whitney *U*-test was used for comparisons between different data sets. Asterisks indicate significant differences (*P < 0.05, **P < 0.01 and ***P < 0.001).

## Supplementary information

**Additional file 1: Figure S1.** Alizarin Red and Alcian Blue staining.

**Additional file 2: Figure S2.** Safranin O staining.

**Additional file 3: Figure S3.** Defects in the growth plate of *Col2cre*; *Kif5b*^*fl/−*^ mice. (A) Immunostaining of KIF5B on the sections of the proximal tibial growth plate of P1 newborns. KIF5B is absent in the majority of the growth plate chondrocytes (indicated area). Scale bar: 150 μm. (B) RT-PCR analysis of total RNA from mainly the proliferating zones of the growth plates (C: *Kif5b*^*fl/+*^; M: *Col2cre*; *Kif5b*^*fl/−*^) showing the absence of *Kif5a*, *Kif5b* and *Kif5c* in mutant. RT-PCR of total RNA from wild-type mouse brain served as a positive control for amplification of *Kif5a* and *Kif5c*. (C) BrdU-incorporation analysis on the sections of the proximal tibial growth plate of P1 newborns. Scale bar: 150 μm.

**Additional file 4: Figure S4.** KIF5B protein level is reduced in primary chondrocytes isolated from *Col2cre*; *Kif5b*^*fl/−*^ mice. (A) Western blot of KIF5B protein in chondrocytes isolated from growth plates of *Kif5b*^*fl/+*^ and *Col2cre*; *Kif5b*^*fl/−*^ mice for primary culture. (B) Immunofluorescence of KIF5B (green) in primary chondrocytes. Scale bar: 10 μm.

**Additional file 5: Movie S1.** Time-lapse series of a primary chondrocyte from *Kif5b*^*fl/+*^ mice. Frames were acquired every 5 min.

**Additional file 6: Movie S2.** Time-lapse series of a primary chondrocyte from *Col2cre*; *Kif5b*^*fl/−*^ mice showing cytokinetic failure in abscission. Frames were acquired every 5 min.

**Additional file 7: Figure S5.** Failure of maintaining of proper cell polarity in Kif5b deficient chondrocytes. (A) Representative images of GM130 immunofluorescence on the growth plate. In normal proliferative chondrocytes, Golgi apparatus (marked by GM130 with red fluorescence) localizes to one or two sides of the nucleus (marked with DAPI). The cell plane is perpendicular to the longitudinal axis of the growth plate (upper). But in KIF5B depleted chondrocytes, the cells are abnormal shaped. Golgi complex scatters around the cytoplasm, with the cell planes abnormally aligned, compared to the longitudinal axis of the growth plate (lower). (B) Representative images of acetylated-α-tubulin immunofluorescence on the growth plates. It is shown that most normal cells display cilia when stained with the antibody for acetylated-α-tubulin (upper). As well, cilia are preferentially located on the inferior/superior surfaces of the flattened chondrocytes. However, although the epiphyseal chondrocytes in the mutant growth plate are less affected, the proliferative chondrocytes are nearly devoid of cilia, with the acetylated tubulin scattered in the whole cell (lower). Scale bar: 10 μm.

**Additional file 8: Figure S6.** Reduced tubulin intensity in midbody in Kif5b knockdown ATDC5 cells. (A) Immunofluorescence of α-tubulin in sh-ctl and sh-Kif5b cells in late cytokinesis. Yellow arrows denote midbody regions. Scale bar: 10 μm. (B) Quantification of tubulin intensity in midbody in both sh-ctl (*n* = 63 cells from sh-ctl clone #1–3) and sh-Kif5b cells (*n* = 98 cells from sh-Kif5b clone #4, #5 and #8). ***P < 0.0001; two-tailed Mann-Whitney *U*-test. The whisker plot shows median (lines), interquartile range (boxes) and 10% to 90% percentile (whiskers).

**Additional file 9: Figure S7.** Localization of MKLP1 or CIT-K is not affected in KIF5B deficient cells. (A) Immunostaining of MKLP1 and CIT-K in primary chondrocytes in late cytokinesis. Red arrows denote the positive signal in midbody regions. Scale bar: 10 μm. (B) Immunostaining of MKLP1 and CIT-K in sh-ctl and sh-Kif5b ATDC5 cells in late cytokinesis. Red arrows denote the positive signal in midbody regions. Scale bar: 10 μm.

**Additional file 10: Figure S8.** Midbody structure in Kif5b knockdown ATDC5 cells is affected. Electron micrographs of sh-ctl (a–f) and sh-Kif5b (a’–f’) cells in cytokinesis. Scale bar: 0.5 μm.

**Additional file 11: Movie S3.** Confocal time-lapse series of a sh-ctl ATDC5 cell expressing GFP-tubulin. Frames were acquired every 1 min.

**Additional file 12: Movie S4.** Confocal time-lapse series of a sh-Kif5b ATDC5 cell expressing GFP-tubulin. Frames were acquired every 1 min.

## Data Availability

All data generated or analysed during this study are included in this published article.

## Abbreviations

KIFs: kinesin superfamily proteins
KIF3A: kinesin superfamily protein 3A
KIF5B: kinesin superfamily protein 5B
KIF5C: kinesin superfamily protein 5C
CIT-K: citron kinase
MKLP: mitotic kinesin-like protein
Cyk-4: cytokinesis 4
PLK1: serine/threonine-protein kinase, also known as polo-like kinase 1
MMP-1: Matrix metalloproteinase-1
