# Supplementary figures and images for: KIF5B modulates central spindle organization in late-stage cytokinesis in chondrocytes
Source: Cell Biosci. 2019 Oct 16;9:85. doi: 10.1186/s13578-019-0344-5 (PMC6794761; doi:10.1186/s13578-019-0344-5)

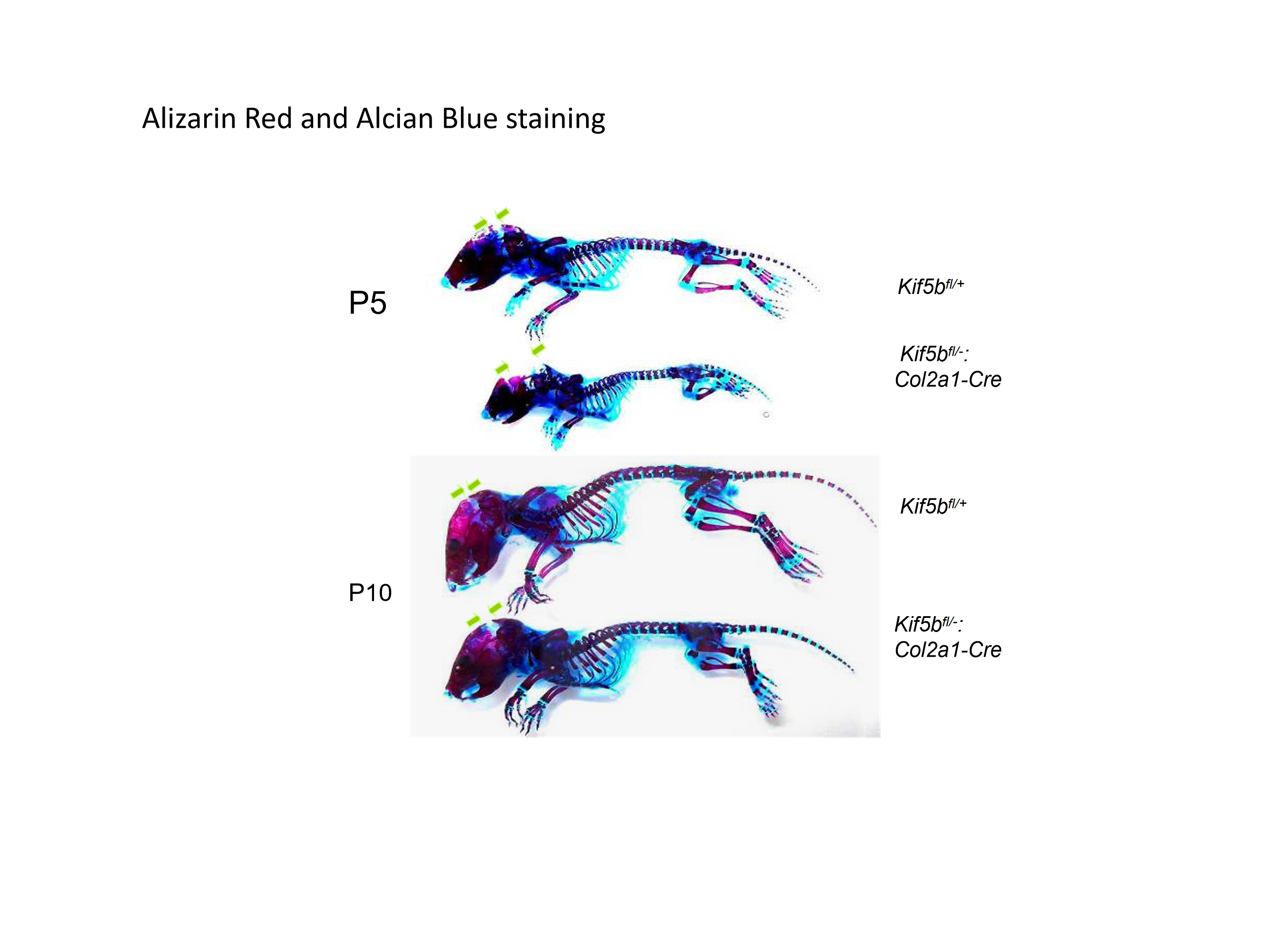

Supplement: Supplementary file 1 — Additional file 1: Figure S1. Alizarin Red and Alcian Blue staining. [file 13578_2019_344_MOESM1_ESM.tif]

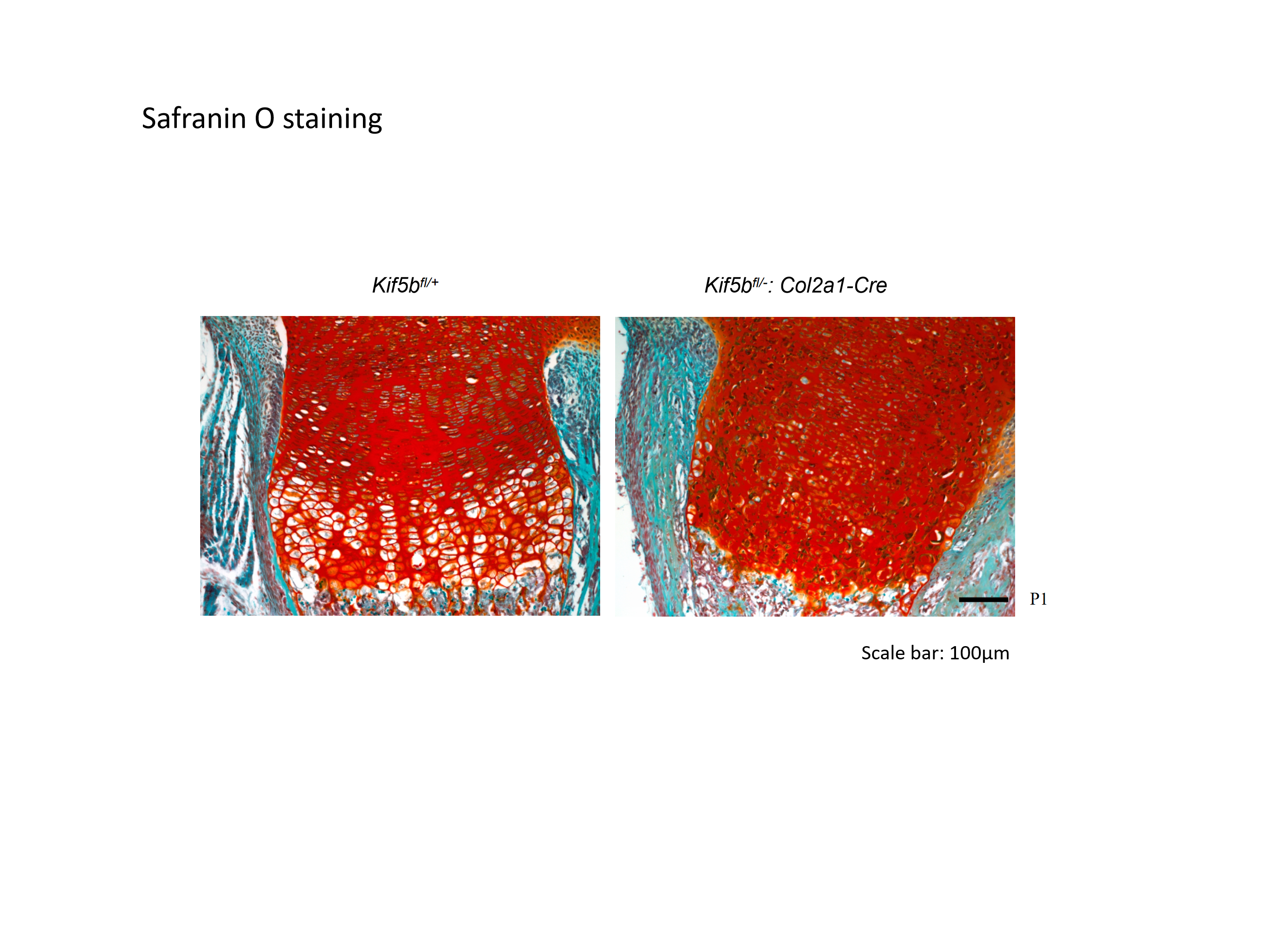

Supplement: Supplementary file 2 — Additional file 2: Figure S2. Safranin O staining. [file 13578_2019_344_MOESM2_ESM.tif]

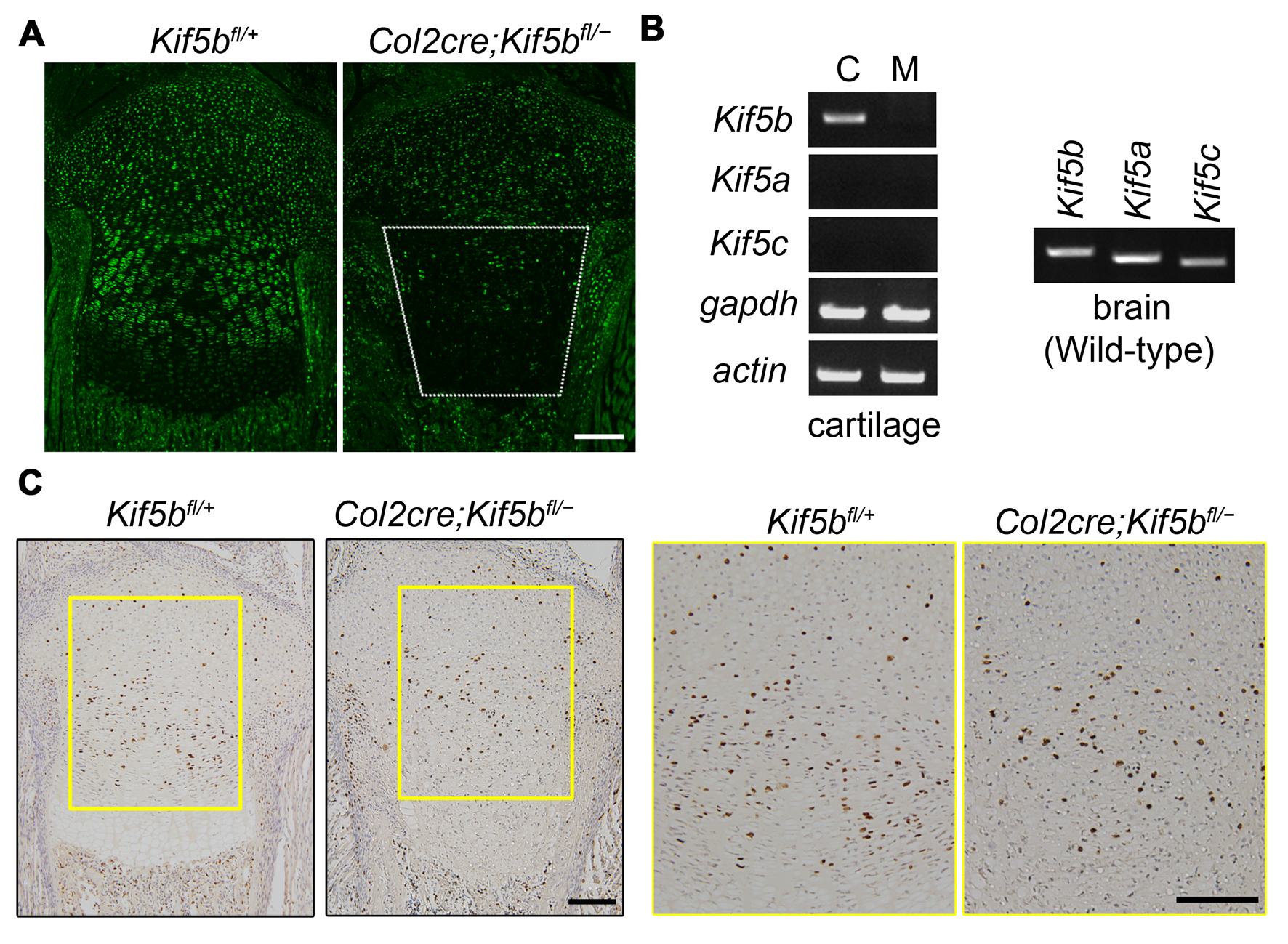

Supplement: Supplementary file 3 — Additional file 3: Figure S3. Defects in the growth plate of Col2cre; Kif5bfl/− mice. (A) Immunostaining of KIF5B on the sections of the proximal tibial growth plate of P1 newborns. KIF5B is absent in the majority of the growth plate chondrocytes (indicated area). Scale bar: 150 μm. (B) RT-PCR analysis of total RNA from mainly the proliferating zones of the growth plates (C: Kif5bfl/+; M: Col2cre; Kif5bfl/−) showing the absence of Kif5a, Kif5b and Kif5c in mutant. RT-PCR of total RNA from wild-type mouse brain served as a positive control for amplification of Kif5a and Kif5c. (C) BrdU-incorporation analysis on the sections of the proximal tibial growth plate of P1 newborns. Scale bar: 150 μm. [file 13578_2019_344_MOESM3_ESM.tif]

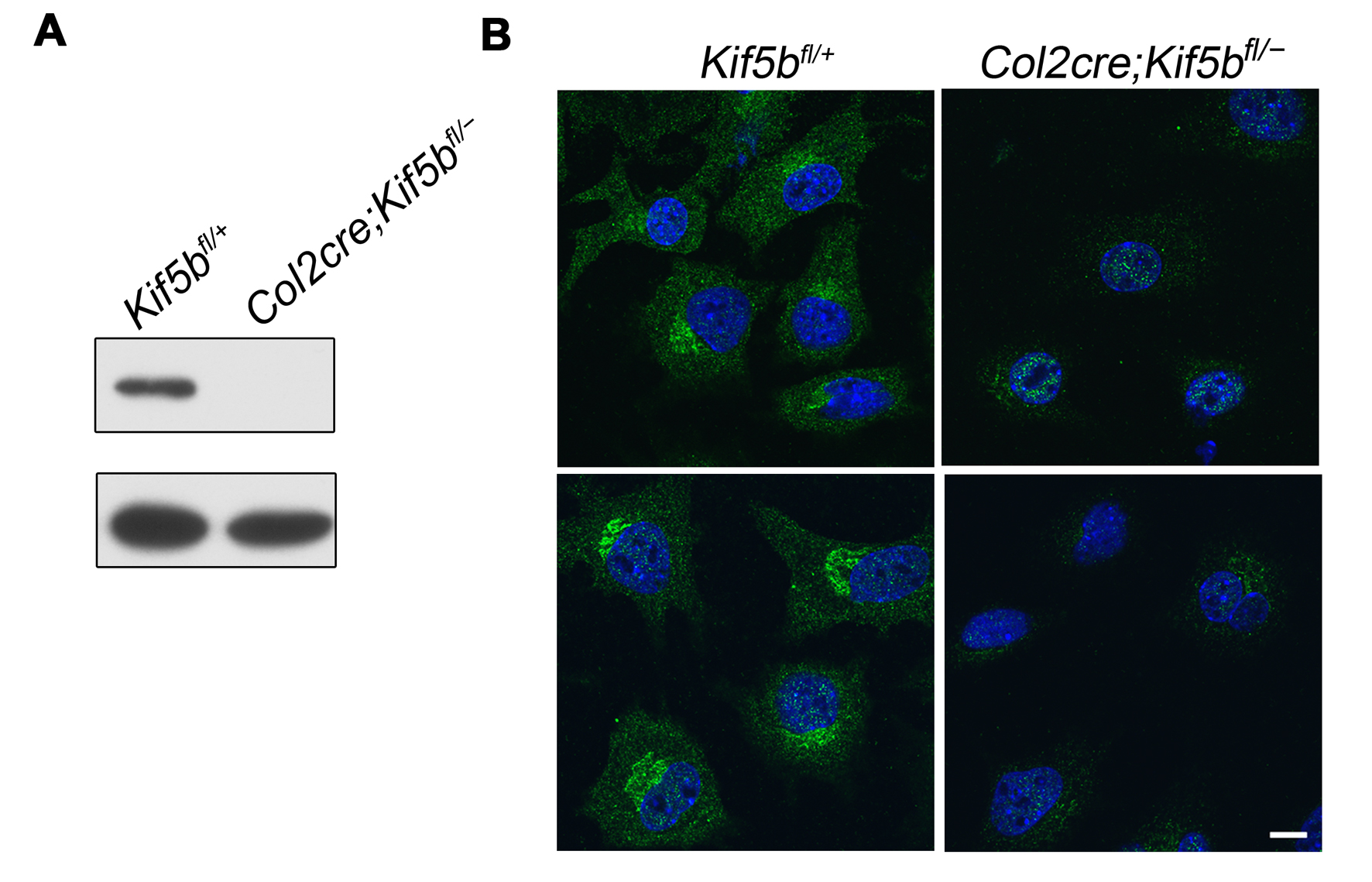

Supplement: Supplementary file 4 — Additional file 4: Figure S4. KIF5B protein level is reduced in primary chondrocytes isolated from Col2cre; Kif5bfl/− mice. (A) Western blot of KIF5B protein in chondrocytes isolated from growth plates of Kif5bfl/+ and Col2cre; Kif5bfl/− mice for primary culture. (B) Immunofluorescence of KIF5B (green) in primary chondrocytes. Scale bar: 10 μm. [file 13578_2019_344_MOESM4_ESM.tif]

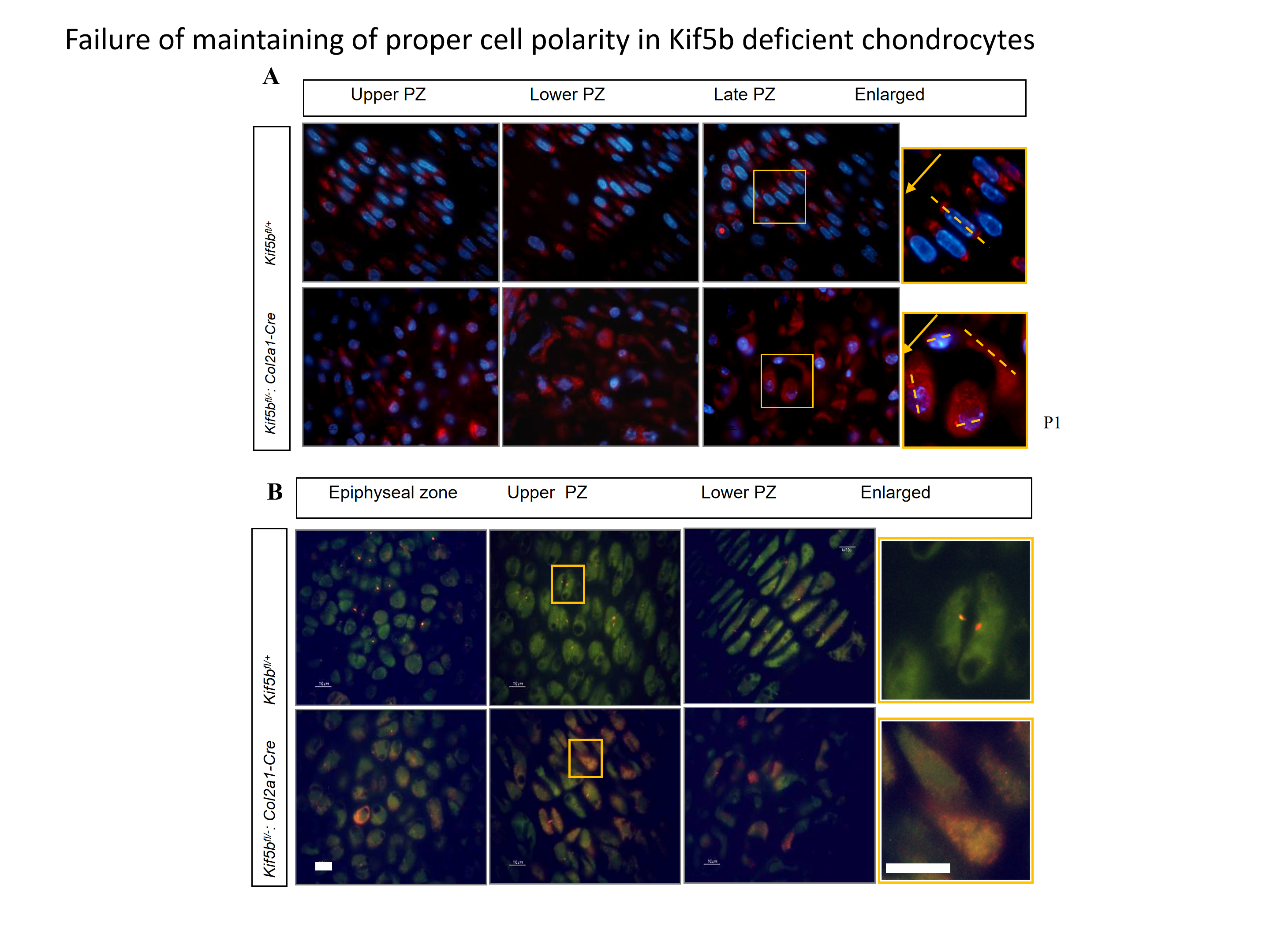

Supplement: Supplementary file 7 — Additional file 7: Figure S5. Failure of maintaining of proper cell polarity in Kif5b deficient chondrocytes. (A) Representative images of GM130 immunofluorescence on the growth plate. In normal proliferative chondrocytes, Golgi apparatus (marked by GM130 with red fluorescence) localizes to one or two sides of the nucleus (marked with DAPI). The cell plane is perpendicular to the longitudinal axis of the growth plate (upper). But in KIF5B depleted chondrocytes, the cells are abnormal shaped. Golgi complex scatters around the cytoplasm, with the cell planes abnormally aligned, compared to the longitudinal axis of the growth plate (lower). (B) Representative images of acetylated-α-tubulin immunofluorescence on the growth plates. It is shown that most normal cells display cilia when stained with the antibody for acetylated-α-tubulin (upper). As well, cilia are preferentially located on the inferior/superior surfaces of the flattened chondrocytes. However, although the epiphyseal chondrocytes in the mutant growth plate are less affected, the proliferative chondrocytes are nearly devoid of cilia, with the acetylated tubulin scattered in the whole cell (lower). Scale bar: 10 μm. [file 13578_2019_344_MOESM7_ESM.tif]

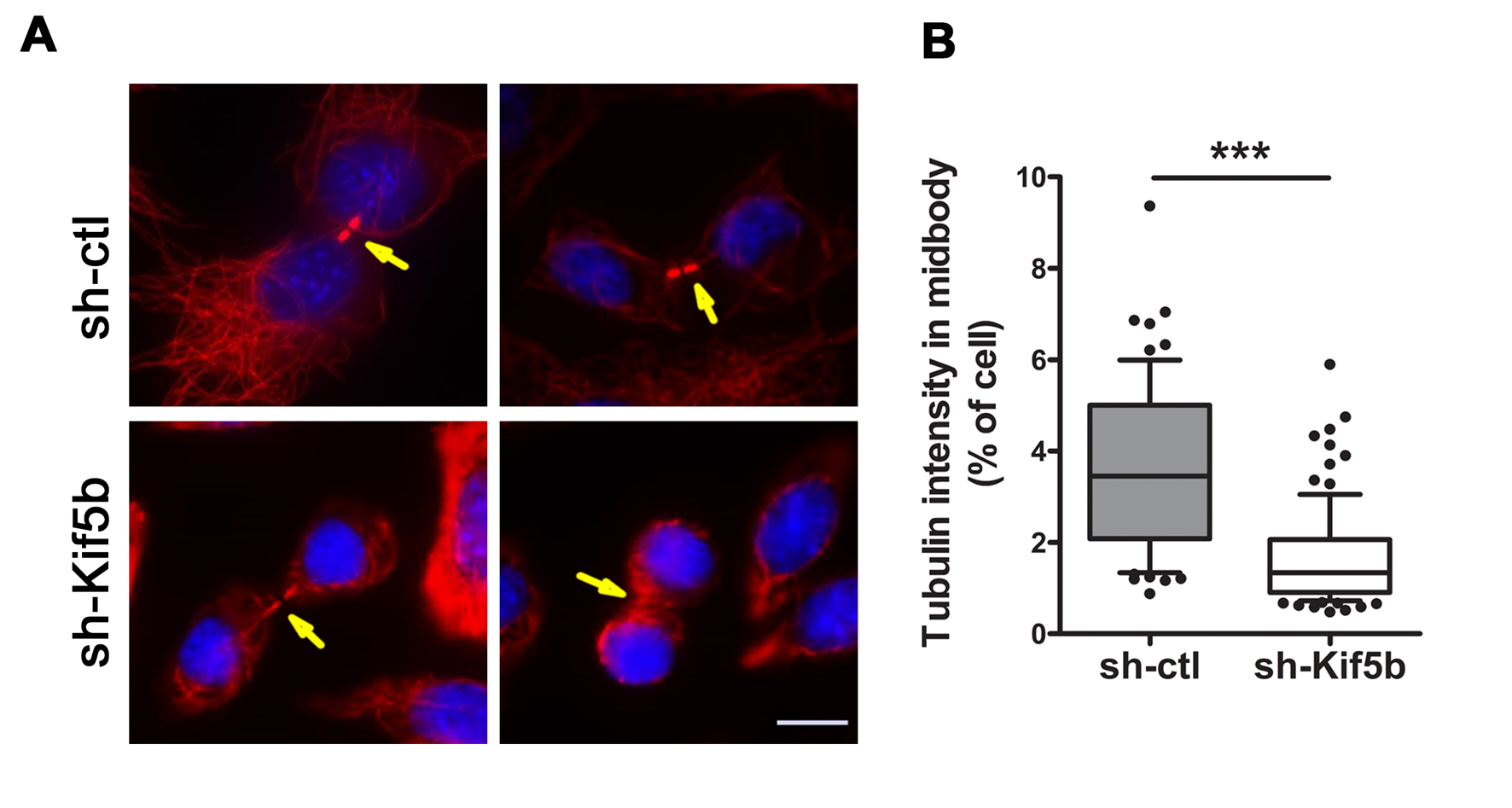

Supplement: Supplementary file 8 — Additional file 8: Figure S6. Reduced tubulin intensity in midbody in Kif5b knockdown ATDC5 cells. (A) Immunofluorescence of α-tubulin in sh-ctl and sh-Kif5b cells in late cytokinesis. Yellow arrows denote midbody regions. Scale bar: 10 μm. (B) Quantification of tubulin intensity in midbody in both sh-ctl (n = 63 cells from sh-ctl clone #1–3) and sh-Kif5b cells (n = 98 cells from sh-Kif5b clone #4, #5 and #8). ***P < 0.0001; two-tailed Mann-Whitney U-test. The whisker plot shows median (lines), interquartile range (boxes) and 10% to 90% percentile (whiskers). [file 13578_2019_344_MOESM8_ESM.tif]

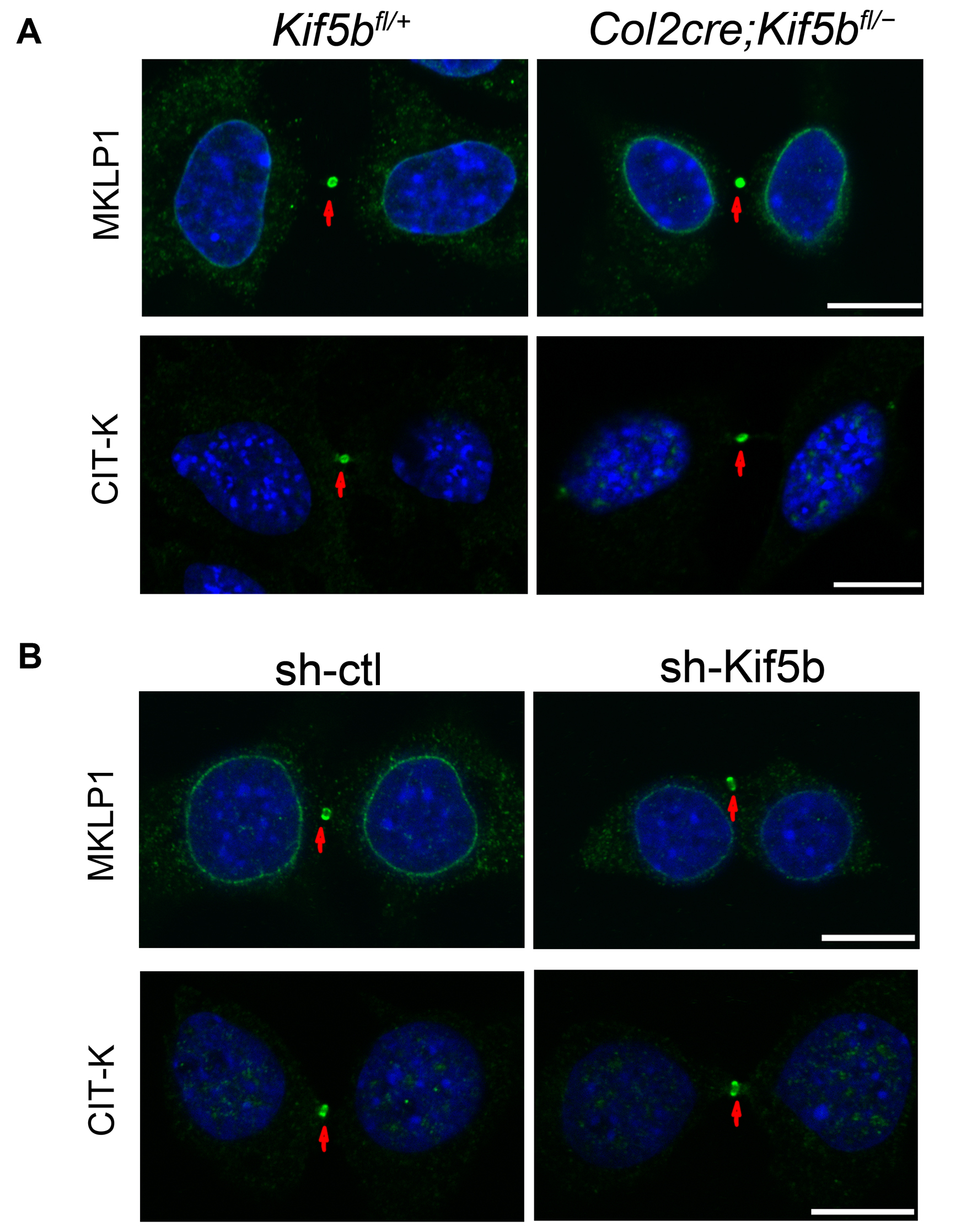

Supplement: Supplementary file 9 — Additional file 9: Figure S7. Localization of MKLP1 or CIT-K is not affected in KIF5B deficient cells. (A) Immunostaining of MKLP1 and CIT-K in primary chondrocytes in late cytokinesis. Red arrows denote the positive signal in midbody regions. Scale bar: 10 μm. (B) Immunostaining of MKLP1 and CIT-K in sh-ctl and sh-Kif5b ATDC5 cells in late cytokinesis. Red arrows denote the positive signal in midbody regions. Scale bar: 10 μm. [file 13578_2019_344_MOESM9_ESM.tif]

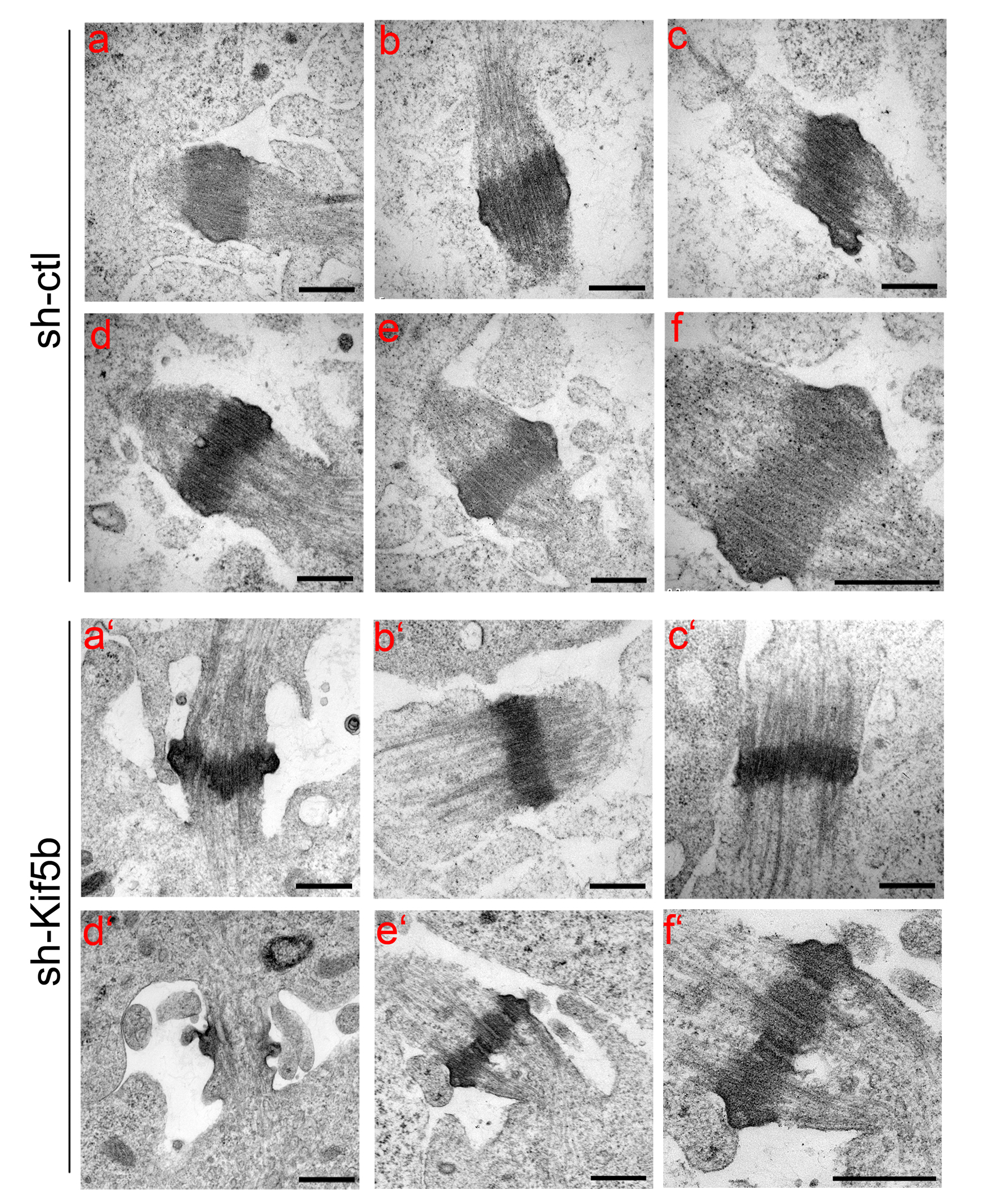

Supplement: Supplementary file 10 — Additional file 10: Figure S8. Midbody structure in Kif5b knockdown ATDC5 cells is affected. Electron micrographs of sh-ctl (a–f) and sh-Kif5b (a’–f’) cells in cytokinesis. Scale bar: 0.5 μm. [file 13578_2019_344_MOESM10_ESM.tif]
